# Hepatic cellular stress response pathways exhibit species differences in basal and inducible activity

**DOI:** 10.1093/toxsci/kfag061

**Published:** 2026-05-28

**Authors:** Hannah Coghlan, Sophie Regan, Bhavik Chouhan, Dominic P Williams, Rowena Sison-Young, Andrew R Jones, Ian M Copple

**Affiliations:** Department of Pharmacology & Therapeutics, Institute of Systems, Molecular & Integrative Biology, University of Liverpool, Liverpool L69 3GE, United Kingdom; Clinical Pharmacology and Safety Sciences, R&D, AstraZeneca, Cambridge CB2 0AA, United Kingdom; Clinical Pharmacology and Safety Sciences, R&D, AstraZeneca, Mölndal SE-431 83, Sweden; Clinical Pharmacology and Safety Sciences, R&D, AstraZeneca, Cambridge CB2 0AA, United Kingdom; Human Liver Research Facility, Faculty of Health & Life Sciences, University of Liverpool, Liverpool L69 3GE, United Kingdom; Department of Biochemistry, Cell and Systems Biology, Institute of Systems, Molecular & Integrative Biology, University of Liverpool, Liverpool L69 3GE, United Kingdom; Department of Pharmacology & Therapeutics, Institute of Systems, Molecular & Integrative Biology, University of Liverpool, Liverpool L69 3GE, United Kingdom; Human Liver Research Facility, Faculty of Health & Life Sciences, University of Liverpool, Liverpool L69 3GE, United Kingdom

**Keywords:** Preclinical species, oxidative stress response, unfolded protein response, autophagy, drug-induced liver injury

## Abstract

Cellular stress response pathways such as the nuclear factor erythroid 2-related factor 2 (NRF2) oxidative stress response, endoplasmic reticulum (ER) stress response, and macroautophagy afford protection against many forms of drug toxicity, including the liver toxicity associated with the formation of reactive drug metabolites. In many cases, clinical drug-induced liver injury is poorly predicted by preclinical toxicology studies. To maximize the translatability of preclinical toxicology studies and inform species selection, we have investigated the relative hepatic stress response capacities of humans and preclinical animal species commonly used in toxicology testing. In control liver tissue, the basal gene and protein expression of stress response pathway components was found to be greater in rodents than nonrodent preclinical species and humans. In addition, following *in vitro* exposure to pharmacological modulators of autophagy and the NRF2 and ER stress responses, rodent hepatocytes generally displayed a greater capacity, relative to those of nonrodent preclinical species and humans, for adaptation to cellular stress. In all, our results indicate that rodent preclinical species possess a greater basal and adaptive hepatic capacity for mitigation of chemical insult than nonrodent preclinical species and humans. This study represents the first to provide a comprehensive comparison of stress response pathway capacity of humans and the animal species most commonly used for preclinical drug safety assessment. Our findings can be used to inform the selection of species for safety testing of drugs with a liability for reactive metabolite-mediated liver toxicity, and to interpret the findings of such studies.

There is a clear aspiration that the drug discovery and development process will transition away from a reliance on *in vivo* animal studies, due to ethical and translational concerns (Wadman 2023; US Food and Drug Administration 2025). Yet, at present, almost all drug development programs use *in vivo* animal studies to assess the toxicity liabilities of a new drug (Sewell et al. 2024). Despite this, animal studies predict human drug-induced liver injury (DILI) only marginally more accurately than random chance (55% to 57% predictive rate) (Olson et al. 2000; Tamaki et al. 2013).

Differences in the activity, specificity, and tissue distribution of drug metabolizing enzymes (Baillie and Rettie 2011) and transporter proteins (Wang et al. 2015), drug target structure and expression (Eastwood et al. 2010), and immunology (Bjornson-Hooper et al. 2022) between humans and preclinical species have previously been shown to affect species sensitivity to drug toxicity. For example, preclinical detection of the mitotoxic potential of fialuridine was impeded by species-specific differences in subcellular localization of the equilibrative nucleoside transporter 1, which resulted in fatal human DILI in clinical trials (McKenzie et al. 1995; Lee et al. 2006). Additionally, the antidiabetic drug troglitazone was withdrawn from the market due to severe clinical hepatotoxicity that was not predicted during preclinical toxicology studies due to species-specific differences in drug exposure and clearance (Smith 2003; Shen et al. 2012).

Many hepatotoxicants exert their effects via chemically reactive metabolites (CRMs), which cause widespread cellular injury via nonspecific covalent interactions with macromolecules, including nucleic acids, proteins, and lipid bilayers (Attia 2010). Several stress responses, including the nuclear factor erythroid 2-related factor 2 (NRF2)-mediated response to oxidative stress and the unfolded protein response (UPR) to endoplasmic reticulum (ER) stress, as well as autophagy, have been shown to afford protection against the DILI caused by many drugs and their CRMs, including acetaminophen (APAP), diclofenac, amiodarone, and isoniazid (Liu et al. 2013; Fredriksson et al. 2014; Zhang et al. 2016; Wandrer et al. 2020). Therefore, differences between humans and preclinical animal species in the capacity to respond to and ameliorate chemical insult via these pathways could contribute to a species’ relative sensitivity to certain forms of DILI.

We recently reported that, upon challenge with equivalent chemical insult resulting from administration of APAP, the rat exhibits a greater basal and adaptive capacity for hepatic stress responses compared with the mouse (Russomanno et al. 2023). However, the true extent of inter-species differences in stress response capacity is relatively unknown, with current literature focusing only on rodent preclinical species and lacking a human comparator, which is necessary to establish translatability. To address this knowledge gap, the present study utilized liver tissue and primary hepatocytes from humans—as the benchmark species—and a broader range of relevant preclinical species, to assess inter-species differences in hepatic stress response capacity. Our results show that rodent preclinical species possess a generally greater capacity for stress response induction than humans and nonrodent preclinical species, which may afford these species reduced sensitivity to CRM-mediated DILI. As such, these results highlight a major limitation of preclinical animal species and reinforce the need for reduced reliance on these models.

## Materials and methods

Unless otherwise stated, materials were purchased from Thermo Fisher Scientific (Waltham, Massachusetts, United States). Torin1, bafilomycin A1 (BA1), and thapsigargin were purchased from Selleckchem (Planegg, Germany), whereas bardoxolone methyl ester (CDDO-Me) and Ki696 were purchased from Sigma (St. Louis, Missouri, United States). Bioinformatic analyses were conducted in RStudio using R version 4.4.1.

### Liver tissue

Human liver tissue samples were accessed via the Human Liver Research Facility at the University of Liverpool. Tissue from patients undergoing planned liver resections was obtained with full written, informed consent by qualified medical staff at Aintree University Hospital (Liverpool, United Kingdom) and stored at −80 °C until use. The study protocol was approved by the National Health Service North West Liverpool Central Research Ethics Committee (11/NW/0327) and adhered to the 1975 Declaration of Helsinki and 2008 Declaration of Istanbul. Liver tissues from untreated rodents were kindly provided by members of research groups within the University of Liverpool (Liverpool, United Kingdom). Liver tissues from vehicle-treated cynomolgus macaques and beagle dogs were provided by Charles River Laboratories (Edinburgh, United Kingdom). Full details of the patients and animals from which tissue samples were obtained are presented in Tables S1 and S2. It should be noted that, while human tissue samples were collected from “healthy” portions of the liver obtained during resection and the donors had not received neoadjuvant chemotherapy, all patients had received a diagnosis of liver cancer. This, therefore, should be considered when comparing to the livers of healthy, untreated animals.

### Primary hepatocyte culture

Cryopreserved primary human (see Table S3 for details of donors), cynomolgus macaque, and beagle dog hepatocytes were obtained from Primacyt (Schwerin, Germany). CD1 mouse and Sprague-Dawley rat CryostaX hepatocytes were obtained from SEKISUI Xenotech (Kansas City, Kansas, United States). To minimize the effect of differential hepatocyte culture procedures on stress response activity, cells were thawed, plated, and cultured according to the same protocol. Specifically, cells were cultured in Corning BioCoat Collagen I-coated plates (Corning, Flintshire, United Kingdom) in Williams Medium E supplemented with 2 mM L-glutamine (Sigma), 100 nM dexamethasone (Sigma), 1× insulin–transferrin–selenium, and 100 U/ml penicillin/100 µg/ml streptomycin (Sigma). For all experiments, media was also supplemented with 10% (v/v) fetal bovine serum to enable proper autophagic function (González-Rodríguez et al. 2014; Spormann et al. 2020) and to prevent aberrant basal activation of autophagy affecting other stress response pathways with which it is integrated (Kalinin et al. 2023). Culture medium was replaced at 6 and 24 h post-plating, at which time cells were exposed to positive control modulators of the stress response pathways under investigation, or 0.5% (v/v) DMSO. Hepatocytes were exposed to the NRF2 activators CDDO-Me and Ki696, or the ER stress inducer, thapsigargin, for 8 or 24 h. Where appropriate, hepatocytes were exposed to the autophagic flux inhibitor BA1 for 2 h, after which they were treated with Torin1 alone or in combination with BA1 for 2 to 8 h. Hepatocyte morphology was monitored by light microscopy to ensure the retention of differentiation throughout the experiment (Fig. S1).

### Western blotting

Briefly, radioimmunoprecipitation assay buffer (Sigma) was used for snap-frozen liver tissue homogenization and hepatocyte lysis. Western blotting was performed as previously described (Russomanno et al. 2023). Table S4 details the antibodies used in this study. NCBI Protein BLAST (Camacho et al. 2009) was used to confirm that the immunogen of each antibody displayed high homology (>85%) with the target protein of each species of interest, indicating a comparable antibody affinity across species (Table S5). Protein expression was quantified using Image Lab version 6.1 (Bio-Rad). Target protein normalization was performed using β-actin or total protein expression (Ponceau S signal) (Moritz 2017).

### RNA sequencing

RNA was isolated from hepatocytes using the Monarch Total RNA Miniprep Kit (New England Biolabs, Massachusetts, United States) in accordance with the manufacturer’s protocol, including an on-column DNase digestion. RNA-seq analysis was performed by Eurofins Genomics LLC (Konstanz, Germany), and reads aligned to the appropriate reference genome. As low-read-count genes are less accurately quantified (Islam et al. 2014), genes with fewer than 30 reads per kilobase per million mapped reads (RPKM) in all samples were excluded. All RNA-seq data were deposited in the NCBI Gene Expression Omnibus (GEO) and are accssible via the accession number GSE330151 (https://www.ncbi.nlm.nih.gov/geo/query/acc.cgi?acc=GSE330151). Differential gene expression analysis was performed using the DESeq2 package (version 1.44.0) (Love et al. 2014). Genes were considered to be significantly changed when a false discovery rate-adjusted *P* value (*p*_adj_) ≤ 0.05 and fold change ≤−1.5 or ≥1.5 were achieved. Heatmaps were generated using the pheatmap package (version 1.0.12) (Kolde 2019). Gene set enrichment analysis (GSEA) was performed using the Bioconductor package clusterProfiler (version 4.12.6) (Yu et al. 2012; Wu et al. 2021). Genome-wide annotation of human, mouse, rat, and dog genes was performed using the packages “org.Hs.eg.db” (version 3.19.1), “org.Mm.eg.db” (version 3.19.1), “org.Rn.eg.db” (version 3.19.1), and “org.Cf.eg.db” (version 3.19.1), respectively (Carlson 2019a, 2019b, 2019d, 2024). As no package was available for annotation of cynomolgus macaque genes, the rhesus macaque (“org.Mmu.eg.db,” version 3.19.1) package was used instead (Carlson 2019c). Gene ontology (GO) biological processes with *P *≤ 0.05 and normalized enrichment score (NES) ≤−1.5 or ≥1.5 were considered to be significantly underrepresented or enriched, respectively. Unadjusted *P* values were reported from GSEA to enable exploration of potentially relevant biological effects of treatments that elicited subtle transcriptional responses in some species, which are obscured by stringent multiple comparison corrections. The Bioconductor package rrvgo (version 1.16.0) was used to reduce GO term redundancy into encompassing parent terms based on semantic similarity (Sayols 2023).

### Analysis of public RNA-seq data

Basal hepatic RNA-seq datasets for CD1 mice, Sprague-Dawley rats, cynomolgus macaques, and beagle dogs (*n* = 3/sex) were obtained from Krause et al. (2023) (GEO: GSE219045). Human hepatic RNA-seq data (*n* = 262) was obtained from the GTEx Portal on June 10, 2025 (Stanfill and Cao 2021). One-to-one human orthologs were mapped onto each species’ gene set using Ensembl BioMart (Dyer et al. 2025). The resulting datasets were combined to yield a final set of 11,074 protein-coding genes with one-to-one orthologs in all species of interest. This gene set was then normalized via ranking mean RPKM to enable inter-species comparison. This dataset was filtered by lists of stress response-associated genes obtained from Qiagen Ingenuity Pathway Analysis (IPA) (Krämer et al. 2014).

### Statistical analysis and data presentation

Statistical analyses were performed in GraphPad Prism 10. Normal distribution of data was assessed using a Shapiro–Wilk test. Normally distributed data were analyzed using a 2-tailed unpaired Student’s *t*-test or One-Way ANOVA followed by Tukey’s post-hoc test, as appropriate. Where data where nonnormally distributed, a Mann–Whitey *U*-test or Kruskal–Wallis test followed by Dunn’s post-hoc test was used, as appropriate. Inter-species comparisons were performed on ranked basal RNA-seq data using a Friedman test followed by a Conover-Iman post-hoc test. Unless otherwise stated, a statistical significance threshold (*p* or *p*_adj_) of 0.05 was utilized. For ease of interpretation, where gene or protein expression was compared between species, the human name (e.g. *NQO1* or NQO1) was used in figures and description of the results.

## Results

### Species differences in basal hepatic stress response pathway activity

**Fig. 1.**
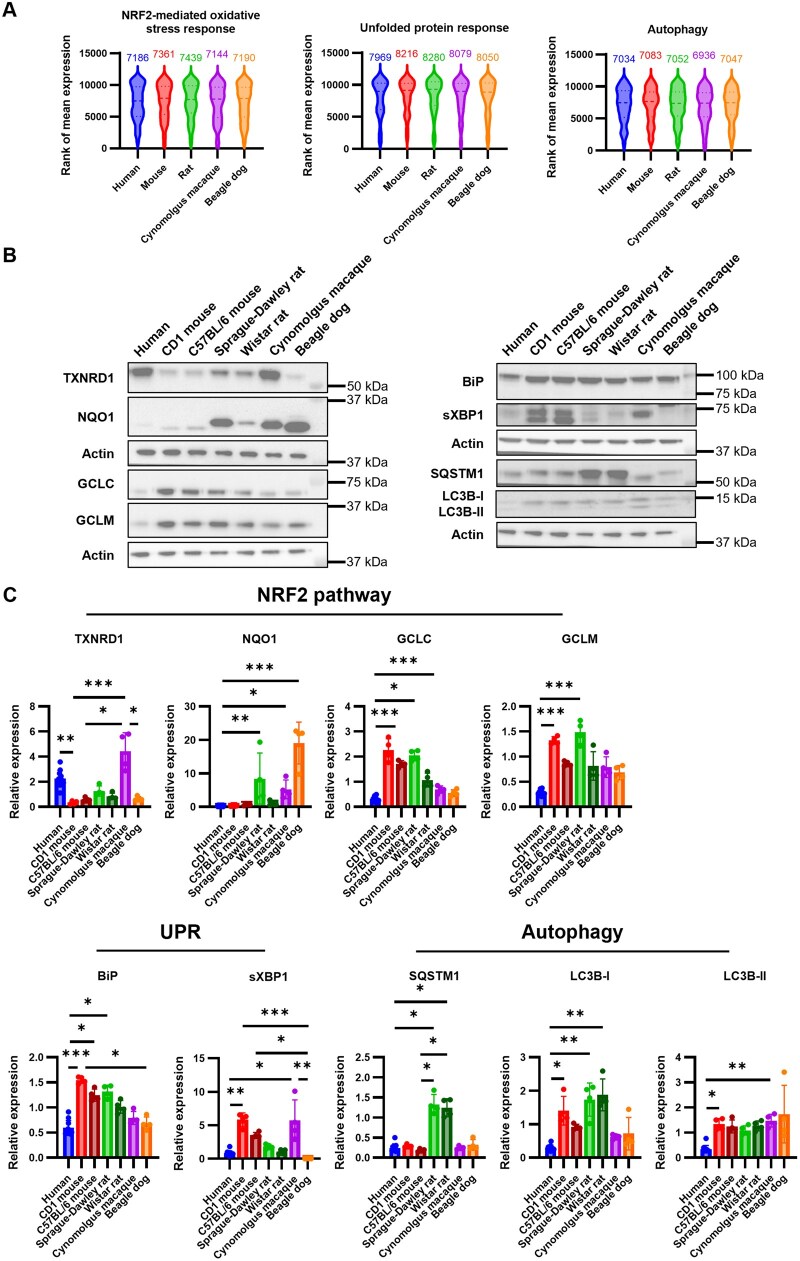
Species differences in basal expression of stress response pathway components in liver. A) Inter-species comparison of the rank of mean expression of genes associated with IPA canonical stress response pathways “NRF2-mediated oxidative stress response” (144 genes), “unfolded protein response” (69 genes), and “autophagy” (167 genes) performed using a Friedman test. Average rank of mean gene expression across all samples per species group is indicated on the graph. B) Western blot visualization and C) densitometric quantification of the expression of stress response-associated proteins in the liver of humans (*n* = 11) and various preclinical animal species (*n* = 4/species). For visualization purposes, samples representative of each group were generated by pooling equal amounts of protein from each biological replicate sample. Target protein expression was normalized to β-actin. Data represent mean ± SD. Inter-species comparisons performed using a Kruskal–Wallis test followed by Dunn’s post-hoc test. Significant inter-species comparisons indicated on the graph: *p_adj_ ≤ 0.05, ** *p*_adj_ ≤ 0.01, *** *p*_adj_ ≤ 0.001.

To assess differences in a species’ capacity to detoxify chemical insult and maintain homeostasis under basal conditions, public liver RNA-seq data—obtained from the human GTEx database (Stanfill and Cao 2021) and the preclinical species gene expression database (Krause et al. 2023)—were leveraged to assess the basal hepatic expression of panels of stress response-associated genes (Fig. 1A). No significant inter-species differences in the basal hepatic expression of NRF2-mediated oxidative stress response, UPR, or autophagy genes were identified, although the mean rank of expression was generally greater in rodents than in nonrodents. To further investigate this, western blotting was used to measure the expression of key stress response components, selected from existing literature, in untreated liver tissues (Fig. 1B). Humans generally displayed the lowest basal hepatic expression of these proteins of all the species investigated (significantly lower expression than at least one preclinical species for 8/9 proteins investigated) (Fig. 1C). In contrast, rodents tended to display the greatest expression of stress response components amongst the preclinical species assessed (Fig. 1C). Overall, the lower expression of key stress response components in human liver may indicate that this species possesses a weaker basal ability to mitigate hepatic chemical insult than the preclinical species investigated, whereas, conversely, preclinical rodent species may possess a relatively enhanced basal capacity for detoxifying chemical insult.

### Investigation of species differences in adaptive stress responses

To support the use of cryopreserved primary hepatocytes as an *in vitro* model for investigating species differences in stress response pathway capacities, inter-species trends in the basal expression of key stress response proteins were compared between snap-frozen liver tissue and cultured primary hepatocytes (Fig. S2). These species-specific trends were found to be generally similar in both models, although the expression of BiP and SQSTM1 were lower in primary hepatocytes than in *ex vivo* liver tissue. These results indicate that, although fundamental differences exist between the models (i.e. the handling, isolation, and cryopreservation of primary hepatocytes, along with the presence of nonparenchymal liver cells in tissue that are absent in isolated hepatocytes), the models are broadly comparable in this respect.

To define treatment conditions for the investigation of species differences in adaptive stress responses, the cytotoxicity and efficacy of the NRF2 activators CDDO-Me and Ki696, the ER stress inducer thapsigargin, and the mTORC1 inhibitor/autophagy inducer Torin1 were assessed *in vitro* (Figs S3 and S4). ATP-based cell viability assays were used to evaluate the cytotoxicity of each compound, whereas RT-qPCR or western blotting were used to assess induction of stress response components. CDDO-Me displayed no cytotoxicity against human and cynomolgus macaque primary hepatocytes *in vitro* but reduced cellular ATP in rodent and beagle hepatocytes at 1 μM (Fig. S3A). As transcriptional induction of the NRF2 pathway by CDDO-Me was time- and concentration-dependent (Fig. S3B), 100 nM CDDO-Me treatment for 24 h was selected. Thapsigargin showed minimal toxicity against cells from most species up to 1 μM, whereas over 50% depletion of ATP content was identified in beagle hepatocytes at concentrations above 30 nM (Fig. S3A). UPR-associated gene induction by thapsigargin was concentration-dependent and maximal at 8 h post-treatment (Fig. S3C). As beagle dog hepatocytes retained approximately 30% viability at 1 μM thapsigargin, while no other species displayed cytotoxicity, this concentration at 8 h exposure was selected for further investigation. Ki696 was screened in HepG2 cells due to the limited availability of primary hepatocytes later in the study (Fig. S3D). Cytotoxicity was observed only following 24 h exposure to 100 μM Ki696; therefore, further experiments were performed using 10 μM Ki696 treatment for 24 h in primary hepatocytes from all species. As autophagy and apoptotic cell death are intrinsically linked (Shliapina et al. 2021), we selected the concentration of the autophagy activator Torin1 based on *in vitro* efficacy alone (Fig. S4). Maximal sequestosome 1 (SQSTM1) degradation and microtubule-associated proteins 1A/1B light chain 3B (LC3B-II) accumulation were identified following 4 h exposure to 3 μM Torin1; hence, this concentration was used for subsequent investigations of autophagic flux.

### Rat hepatocytes exhibit a high capacity for autophagy

**Fig. 2.**
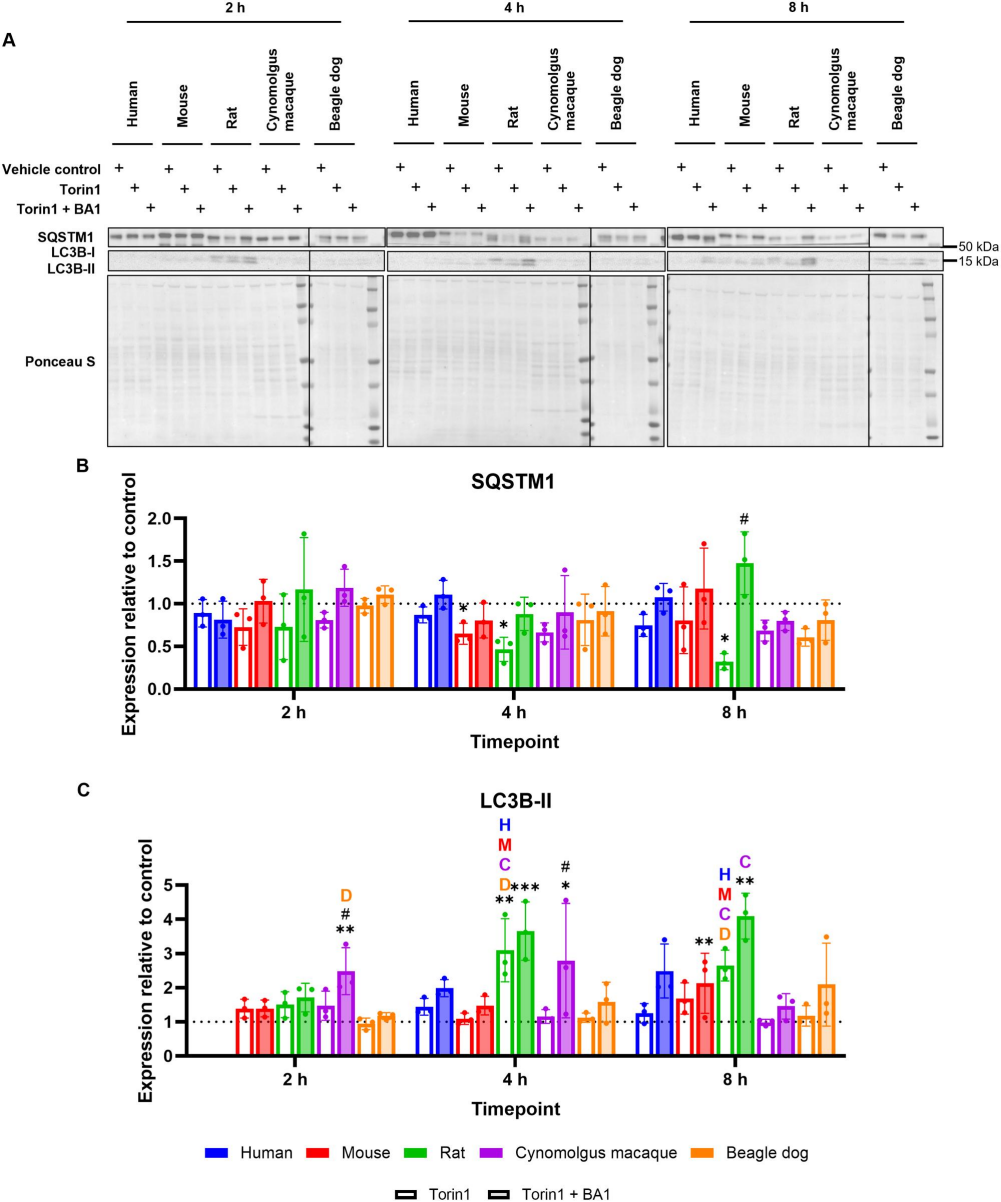
Activation of autophagy in hepatocytes from humans and important preclinical species. A) Western blot visualization and densitometric quantification of (B) SQSTM1 and (C) LC3B-II protein expression in primary hepatocytes following 2, 4, or 8 h exposure to 3 μM Torin1 with or without 2 h pretreatment and cotreatment with 30 nM Bafilomycin A1 (BA1). Target protein expression was normalized to total protein expression. Data represent mean ± SD of *n* = 3 biological replicates. For visualization purposes, samples representative of each group were generated by pooling equal amounts of protein from each biological replicate sample (*n* = 3), where necessary different protein inputs were utilized for different species to ease detectability. Following normality assessment using a Shapiro–Wilk test, differences between treatment groups and species were assessed using a One-Way ANOVA and Tukey’s post-hoc test or Kruskal–Wallis test and Dunn’s post-hoc test, as appropriate. Asterisks denote significant changes relative to vehicle control, hashes denote significant differences between treatment conditions; */# *p*_adj_ ≤ 0.05, **/## *p*_adj_ ≤ 0.01, ***/### *p*_adj_ ≤ 0.001. Letters denote significantly greater induction than the indicated species (*p*_adj_ ≤ 0.05): H, human; M, mouse; C, cynomolgus macaque; D, beagle dog.

Autophagy is primarily regulated by the post-translational modification of its protein machinery, rather than by large-scale transcriptional adaptation as in the case of the NRF2 pathway and UPR. As such, western blot analysis of the expression of SQSTM1 and LC3B is a mainstay method for the assessment of autophagy (Klionsky et al. 2021). To compare species-specific autophagic responses, hepatocytes were exposed to Torin1 with or without BA1, an inhibitor of lysosomal acidification and autophagosome–lysosome fusion, for 2 to 8 h (Fig. 2A).

The expression of LC3B was undetectable in human hepatocytes shortly (i.e. 2 h) following autophagy activation by Torin1, although LC3B was detected in primary hepatocytes from other species at this timepoint and in human hepatocytes at later timepoints (Fig. 2A, C). Autophagic degradation of SQSTM1, albeit nonsignificant, that was partially or wholly restored upon BA1 co-treatment was identified in all species of interest, confirming functional autophagic flux in all species (Fig. 2A, B).

To a greater extent than in the other species, Torin1 treatment resulted in a significant, time-dependent decrease in SQSTM1 protein in rat hepatocytes that was wholly abolished by co-treatment of cells with the autophagic flux inhibitor, BA1, indicating an increased capacity for lysosomal degradation of autophagic cargo in this species (Fig. 2B). Additionally, as shown in Fig. 2C, Torin1 increased the formation of LC3B-II in rat hepatocytes to a significantly greater extent than all other species of interest at both 4 and 8 h post-treatment. This accumulation was further enhanced by BA1 co-treatment, confirming that Torin1-induced LC3B-II elevations reflected increased autophagic flux rather than impaired degradation (Fig. 2C). Significant degradation of SQSTM1 or accumulation of LC3B-II was not identified in cynomolgus macaque hepatocytes following Torin1 treatment at any timepoint. However, Torin1-BA1 co-treatment caused a significant accumulation of LC3B relative to both Torin1 alone and vehicle control at 2 and 4 h post-treatment (Fig. 2C), suggesting high levels of autophagic LC3B-II turnover in cynomolgus macaque cells.

In all, rat hepatocytes, followed by cynomolgus macaque hepatocytes, demonstrated the most pronounced autophagic flux *in vitro* based on both SQSTM1 degradation and LC3B-II accumulation. In contrast, hepatocytes from humans, mice, and beagle dogs exhibited relatively weak responses, which may reflect intrinsic species differences in autophagy capacity.

### Rodent hepatocytes exhibit a strong transcriptional UPR

The UPR restores homeostasis following ER stress via a biphasic adaption involving an initial arrest of global translation that lessens cellular protein folding load, followed by a transcriptional response that promotes protein folding and degradation (Reid et al. 2014). Therefore, to explore inter-species differences in UPR capacity, RNA-seq was employed to quantify changes in hepatocyte mRNA expression caused by the ER stress inducer, thapsigargin.

**Fig. 3.**
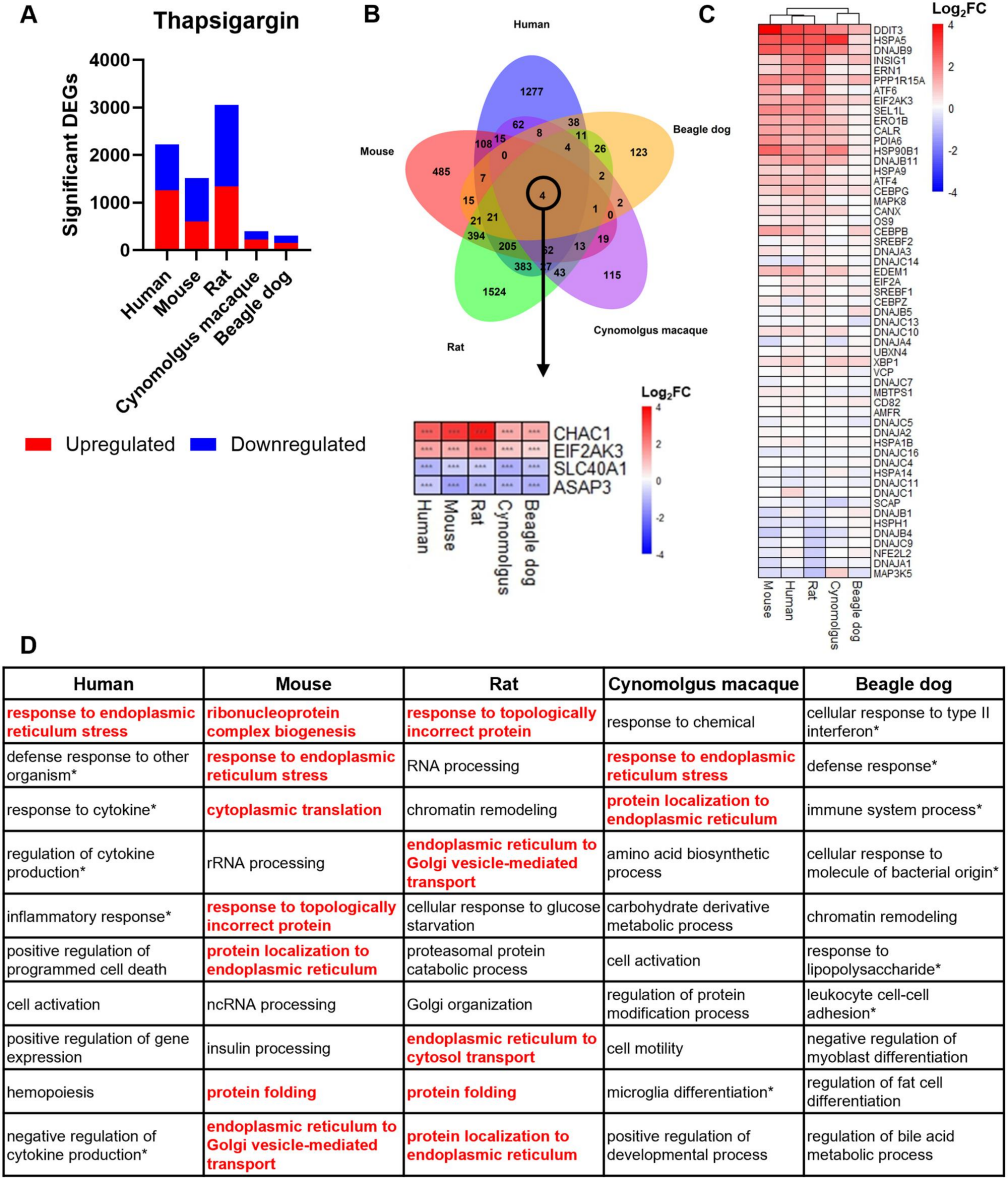
Global transcriptional effects of ER stress induction by thapsigargin in primary hepatocytes. A) Summary of the results of differential gene expression analysis for primary hepatocytes exposed to 1 μM thapsigargin for 8 h, relative to 0.5% (v/v) DMSO vehicle control (*n* = 3/group). B) Identification of genes differentially expressed in primary hepatocytes of all species of interest following thapsigargin exposure. Asterisks denote the significance of the change; *** *p*_adj_ ≤ 0.001. C) Changes in expression of genes associated with the IPA canonical pathway “unfolded protein response” following thapsigargin exposure, relative to vehicle control. D) The top 10 most significantly enriched (NES ≥ 1.5, *P *≤ 0.05) GO parent terms in each species of interest following thapsigargin exposure. Parent terms highly relevant to the UPR are highlighted. Parent terms associated with immune and inflammatory processes are denoted by asterisks.

Global transcriptional adaptation to ER stress was far weaker in primary hepatocytes from the nonrodent preclinical species investigated than in those of humans and preclinical rodent species (Fig. 3A; full list of differentially expressed genes (DEGs) is provided in Supplementary File 1). Almost half of all DEGs were downregulated in each species, which may indicate the induction of regulated inositol-requiring enzyme 1 IRE1-dependent decay, which reduces the translational and folding load of the ER by selectively degrading mRNA (Le Thomas et al. 2021). In all species, many DEGs were species-specific or common to just 2 species, and just 4 genes—*CHAC1*, *EIF2AK3*, *SLC40A1*, and *ASAP3*—were commonly changed in all species of interest (Fig. 3B). As shown in Fig. 3C, assessment of the expression of genes associated with the IPA canonical pathway “unfolded protein response” also confirmed that thapsigargin induced a greater magnitude of transcriptional change in human, mouse, and rat than in macaque and dog, with highly and poorly responsive species clustering separately.

GSEA revealed a large number of significantly changed biological processes in all species of interest (Supplementary File 2), which were summarized into nonredundant parent terms using rrvgo. Fig. 3D summarizes the 10 most enriched GO parent terms in each species of interest. No UPR-associated terms were enriched in beagle dog hepatocytes, in line with the minor transcriptional induction previously identified (Fig. 3A). However, although cynomolgus macaque hepatocytes also displayed a weak global transcriptional response to ER stress induction, both “response to endoplasmic reticulum stress” and “protein localization to endoplasmic reticulum” were highly enriched terms in this species. In contrast, despite human hepatocytes displaying a much stronger global transcriptional adaptation than the nonrodent preclinical species, only 1 relevant term was within the top 10 enriched terms in this species, highlighting a divergence in the inter-species trends identified across different measures of responsiveness. Instead, in human and dog hepatocytes, many of the most enriched pathways were associated with immune and inflammatory processes, which are known to be associated with ER stress responses even under sterile conditions (Garg et al. 2012). However, as expected, processes associated with the ER stress response and protein synthesis, folding, and transport represented most of the top enriched biological processes in mouse and rat hepatocytes (Fig. 3D). To confirm the findings of RNA-seq, targeted RT-qPCR analysis was performed to assess the induction of UPR-associated genes selected from literature (*HSPA5*, *EDEM1*, and spliced *XBP1*; Fig. S5A). Different inter-species trends in transcriptional induction were identified across different UPR targets, highlighting the need for global, rather than targeted, comparisons. However, this approach also showed a greater mRNA induction in rodent hepatocytes and a lack of transcriptional adaptation in beagle dog hepatocytes.

To assess whether the robust transcriptional adaptation of human and rodent hepatocytes to ER stress was conserved at the protein level, the effect of thapsigargin treatment on expression of the UPR regulator BiP (encoded by *HSPA5*) and the key UPR transcription factor XBP1 was assessed by western blotting (Fig. S6). Additionally, the expression and phosphorylation of SAPK/JNK, which occurs under conditions of cell stress, was measured. Thapsigargin induced very few changes in the proteins assessed in this study. The phosphorylation of the 54 kDa isoform of SAPK/JNK in beagle hepatocytes following 8 h thapsigargin treatment (1.34 ± 0.18-fold), although not significant relative to vehicle control, was significantly greater than in hepatocytes from humans (0.95 ± 0.04-fold, *p*_adj_ = 0.0134), mice (0.86 ± 0.18-fold, *p*_adj_ = 0.0038), and cynomolgus macaques (0.93 ± 0.13-fold, *p*_adj_ = 0.0100), which may be associated with the enhanced susceptibility to thapsigargin cytotoxicity of canine hepatocytes.

### Rodent hepatocytes exhibit a strong NRF2-driven oxidative stress response

The cellular response to oxidative stress is primarily regulated by NRF2, a transcription factor that regulates the expression of hundreds of antioxidant and cytoprotective genes (Copple et al. 2019). Under basal conditions, NRF2 is sequestered in the cytosol and repressed by a homodimer of Kelch-like ECH-associated protein 1 (KEAP1) (Iso et al. 2016). Therefore, RNA-seq was utilized to assess transcriptional adaptation to NRF2 activation by the electrophilic inhibitor of KEAP1, CDDO-Me.

**Fig. 4.**
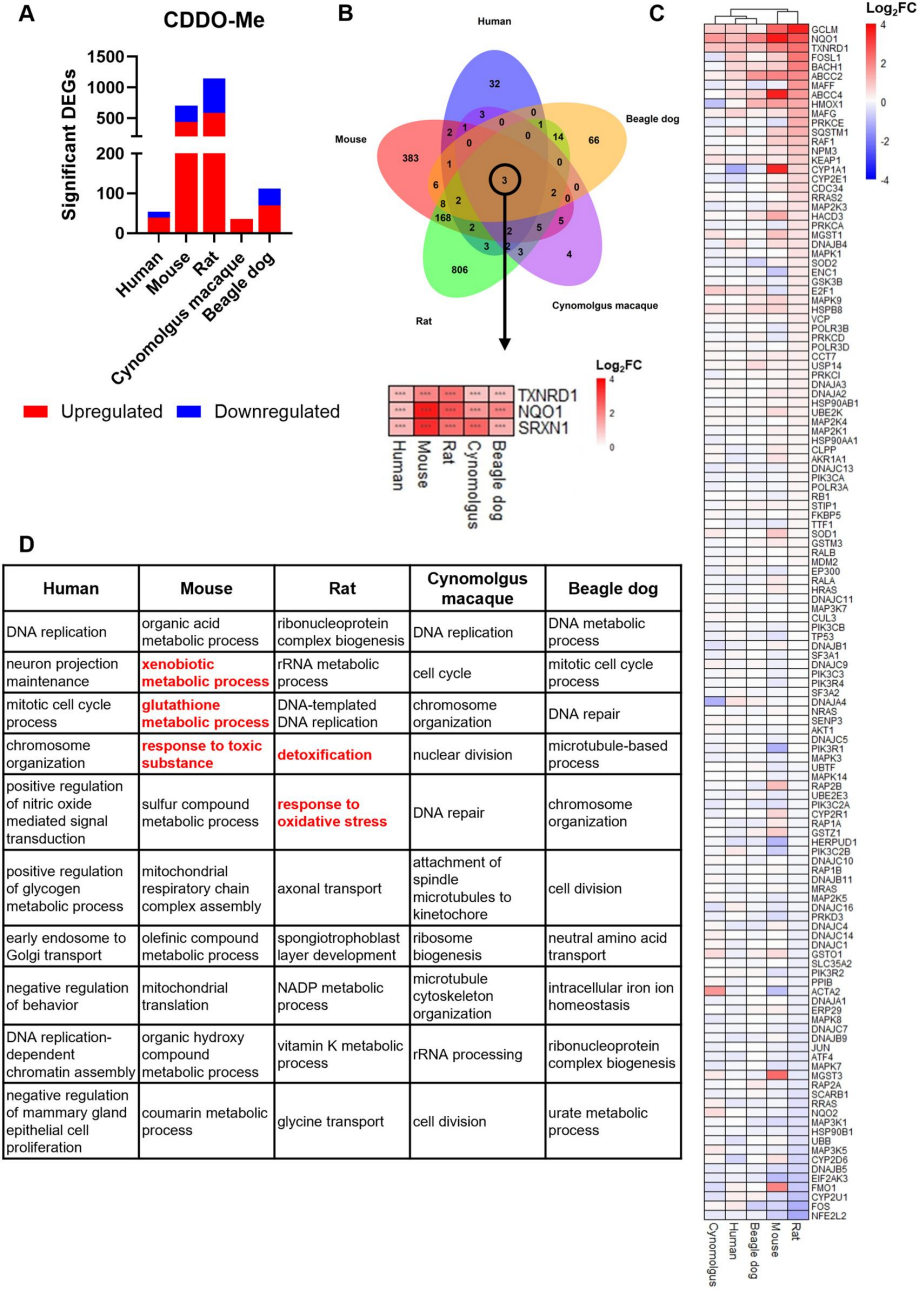
Global transcriptional effects of KEAP1 inhibition by CDDO-Me in primary hepatocytes. A) Summary of the results of differential gene expression analysis for primary hepatocytes exposed to 100 nM CDDO-Me for 24 h, relative to 0.5% (v/v) DMSO vehicle control (*n* = 3/group). B) Identification of genes differentially expressed in primary hepatocytes of all species of interest following CDDO-Me exposure. Asterisks denote the significance of the change; *** *p*_adj_ ≤ 0.001. C) Changes in expression of genes associated with the IPA canonical pathway “NRF2-mediated oxidative stress response” following CDDO-Me exposure, relative to vehicle control. D) The top 10 most significantly enriched (NES ≥ 1.5, *P *≤ 0.05) GO parent terms in each species of interest following CDDO-Me exposure. Parent terms highly relevant to the NRF2 pathway are highlighted.

Following NRF2 activation, the rodent preclinical species investigated mounted far stronger transcriptional responses than humans and the nonrodent preclinical species of interest, as presented in Fig. 4A. In all species except cynomolgus macaque (due to the very minor transcriptional response observed in this species), the majority of DEGs were species specific. Only 3 genes—*NQO1*, *TXNRD1*, and *SRXN1—*were commonly changed in all 5 species of interest, although a greater change in the expression of these genes was identified in rodents than in nonrodents (Fig. 4B). RT-qPCR assessment of the induction of *NQO1*, *TXNRD1*, and *SRXN1* in primary hepatocytes confirmed this trend (Fig. S5B). The complete results of differential gene expression analysis are provided in Supplementary File 3.

Rodents also displayed a greater magnitude of change in the expression of a set of NRF2 pathway-associated genes than nonrodents, with these highly and poorly responsive species groups once again clustering separately due to their differing gene expression patterns (Fig. 4C). Furthermore, as summarized in Fig. 4D, GSEA identified that GO processes associated with the NRF2-mediated antioxidant response, including xenobiotic and glutathione metabolic processes, detoxification, and responses to toxic substances and oxidative stress, were among the most enriched pathways in both rodent species but were absent in humans and the nonrodent preclinical species (Supplementary File 4). Instead, processes associated with DNA replication, chromosome organization, and the cell cycle were most enriched in these species in response to CDDO-Me exposure.

Electrophilic NRF2 activators such as CDDO-Me have been shown to interact with other cellular targets, impacting pharmacological specificity (Yore et al. 2011). To overcome this limitation, inhibitors of the NRF2-KEAP1 protein–protein interaction (PPI) have been developed as more specific activators of the NRF2 response (Tran et al. 2019). Hence, to further explore species differences in NRF2 pathway activity and to evaluate whether they are conserved across different mechanisms of NRF2 activation, transcriptional responses to the PPI inhibitor, Ki696 (Davies et al. 2016), were investigated.

**Fig. 5.**
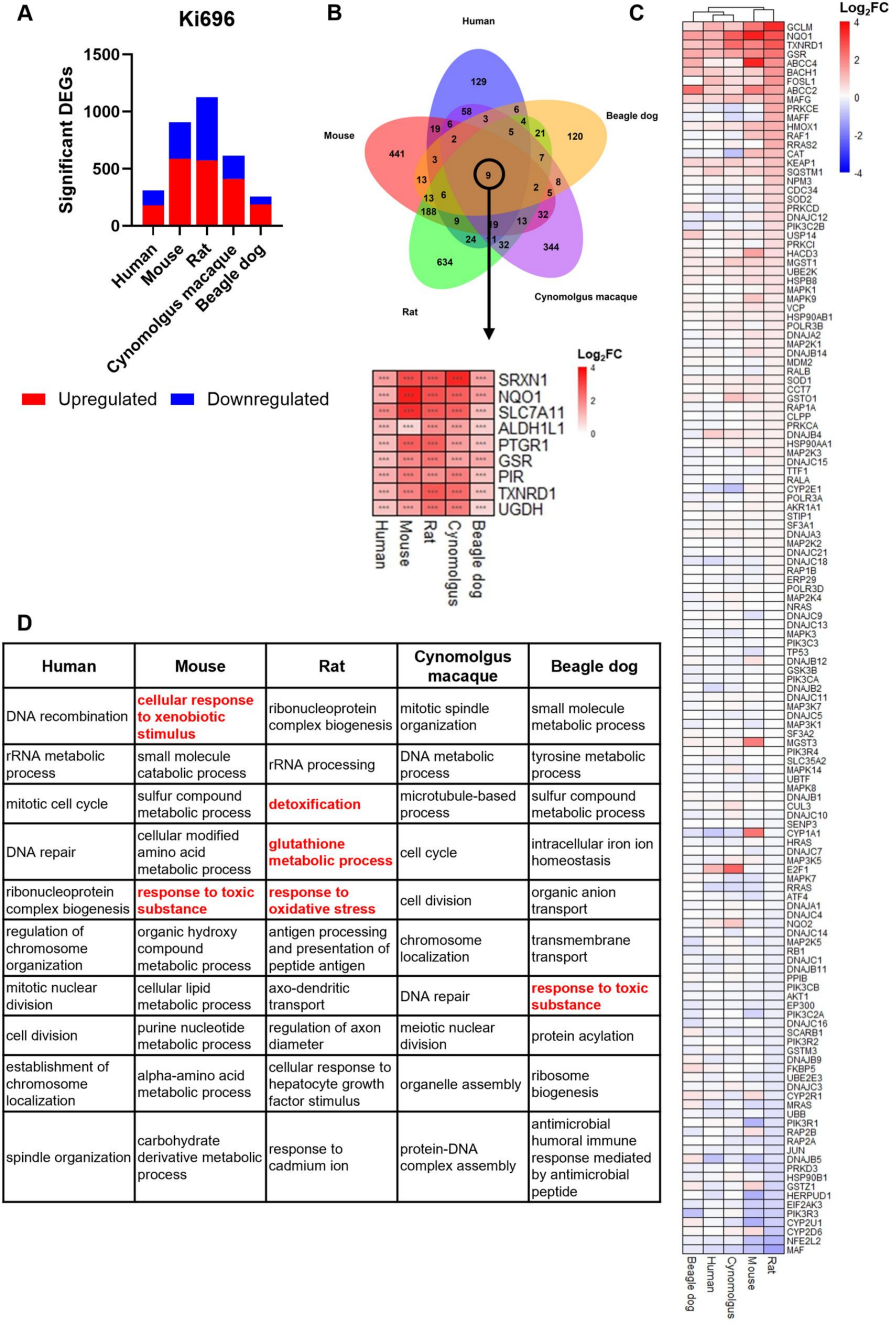
Global transcriptional effects of KEAP1 inhibition by Ki696 in primary hepatocytes. A) Summary of the results of differential gene expression analysis for primary hepatocytes exposed to 10 μM Ki696 for 24 h, relative to 0.5% (v/v) DMSO vehicle control (*n* = 3/group). B) Identification of genes differentially expressed in primary hepatocytes of all species of interest following Ki696 exposure. Asterisks denote the significance of the change; *** *p*_adj_ ≤ 0.001. C) Changes in expression of genes associated with the IPA canonical pathway “NRF2-mediated oxidative stress response” following Ki696 exposure, relative to vehicle control. D) The top 10 most significantly enriched (NES ≥ 1.5, *P *≤ 0.05) GO parent terms in each species of interest following Ki696 exposure. Parent terms highly relevant to the NRF2 pathway are highlighted.

Similar to the effects of CDDO-Me, mouse and rat hepatocytes displayed the greatest global transcriptional responses to Ki696, with weaker responses observed in human, cynomolgus macaque, and beagle dog hepatocytes (Fig. 5A; Supplementary File 5). Most DEGs remained species-specific, but a larger panel of 9 genes was identified to be commonly changed by Ki696 across all species (Fig. 5B). This panel included *NQO1*, *TXNRD1*, and *SRXN1*, which were also upregulated by CDDO-Me in all species, suggesting that this subset of genes may form the most robust and translatable markers of NRF2 pathway activation, supporting previous conclusions by Morgenstern et al. (2024). Targeted assessment of the expression of these genes also confirmed that rodent hepatocytes exhibit stronger NRF2-mediated transcriptional responses than nonrodent hepatocytes (Fig. S5C). Most of the remaining commonly changed DEGs (*SLC7A11*, *PTGR1*, *GSR*, *PIR*, and *UGDH*) were differentially expressed in multiple, but not all, species of interest following CDDO-Me treatment. Also in line with responses to CDDO-Me, rodents displayed a greater activation of the NRF2 pathway by Ki696 compared with nonrodents, as measured by changes in the expression of NRF2-associated genes (Fig. 5C) and the enrichment of NRF2-associated biological processes in GSEA (Fig. 5D; Supplementary File 6). Furthermore, Ki696 caused the enrichment of GO processes associated with DNA replication and the cell cycle in human and cynomolgus macaque hepatocytes, indicating that the activation of these pathways is associated with NRF2 signaling in these *in vitro* models, as opposed to the off-target effects of CDDO-Me.

The conservation of species-specific differences in transcriptional responses to NRF2 activation at the protein level were assessed by western blot analysis of NRF2 pathway components, including 2 of those identified as being most transcriptionally conserved (Figs 4 and 5), namely NQO1 and TXNRD1. More pronounced translational responses were identified following 24 h treatment with CDDO-Me than 8 h treatment, in line with the time-dependence of NRF2 activation identified in Fig. S3. As shown in Fig. S7, at 8 h, significant upregulation of NRF2 pathway proteins was primarily identified in rat hepatocytes, whereas mouse hepatocytes also displayed significant upregulations at 24 h. In contrast, no significant changes in NRF2 proteins were identified in nonrodent species—except HMOX1 in beagle hepatocytes at 24 h (1.26 ± 0.07-fold, *P *= 0.0057), although this change was minor—consistent with the results of RNA-seq (Fig. 4). At 24 h exposure, upregulation of all proteins in rat hepatocytes was significantly greater than in cynomolgus macaque and dog hepatocytes, whereas the upregulation of TXNRD1 and GCLC was also greater than in human and mouse cells, and HMOX1 and NQO1 compared with human cells. In all, although changes in protein expression were of a lower magnitude than changes in NRF2 target genes, the same consistent trend was observed, wherein rodent hepatocytes displayed markedly more robust adaptation than cells from nonrodents.

## Discussion

The detection of hepatotoxicity liabilities during preclinical studies is essential in minimizing patient risk during clinical trials and post-approval use. Until recently, most major drug regulators mandated the use of *in vivo* animal studies in the preclinical safety investigation of a new small molecule drug. However, animal studies poorly predict human DILI (Olson et al. 2000; Tamaki et al. 2013) due to an incomplete understanding of the intrinsic similarities and differences between humans and preclinical model species and the impact on a species’ relative sensitivity to toxicity. As sufficiently predictive and validated animal-free new approach methodologies (NAMs) are not yet widely available, animal studies will likely remain a part of drug safety evaluation for the foreseeable future (Madorran et al. 2020). Hence, there remains a need to improve our understanding of the differences between humans and preclinical species to improve the translatability and predictivity of animal toxicology studies. This study compared the capacities of key DILI-associated stress response pathways in humans and preclinical species. Our findings highlight notable inter-species differences that should be considered during preclinical species selection and the interpretation of preclinical toxicology studies.

We recently reported the effect of differential stress response pathway activation in laboratory mice and rats on the hepatotoxicity of APAP, which exerts its toxicity via a CRM (Russomanno et al. 2023). Our findings were consistent with the work of Xu et al. (2021), who investigated the differential responses of mice and rats to the hepatotoxin psoraleae fructus. Importantly, we accounted for species-specific differences in APAP bioactivation via the determination of APAP doses that generated equivalent CRM burden in each species (Russomanno et al. 2023). In the present study, we expanded the number of species considered and utilized specific, positive-control pharmacological modulators of the relevant pathways to assess stress response activation *in vitro* without confounding factors associated with species-specific bioactivation of a hepatotoxicant or nonspecific interactions of a CRM.

We have found that the rodent preclinical species investigated—particularly rats—display a notably greater hepatic capacity for adaptation to stress response activation than humans and the most commonly used nonrodent preclinical species, cynomolgus macaques, and beagle dogs. In a review of the preclinical and clinical safety of approved drugs, Olson et al. (2000) found that nonrodent preclinical species predict human toxicities far more accurately than rodent preclinical species. The similar basal and adaptive stress response capacities of nonrodent preclinical species and humans identified in this study may contribute to these greater predictive capabilities. In contrast, the robust stress response capacity of rodents—particularly rats—identified in this study may render these species less sensitive than humans to hepatic chemical insult and DILI, contributing to the relatively poor detection of human toxicities by these species (Olson et al. 2000; Monticello et al. 2017). Our findings also suggest that species with more robust stress response capacities (i.e. rodents) may be more resistant to conditions such as aging-associated oxidative damage, starvation-associated liver injury, and tumorigenesis, which are associated with cellular stress or are mitigated by stress response pathway activation (Zhang et al. 2015; Zhou et al. 2022). However, the effect of inter-species differences in stress response capacities on these phenotypes has not yet been investigated. In the future, the scope of our work can be expanded by considering other stress responses implicated in DILI, such as the DNA damage and mitochondrial respiratory responses, as well as other tissues relevant to common drug toxicities. In addition, further mechanistic work is necessary to correlate stress response capacity to DILI sensitivity in relevant species, including additional rodent strains and NHP species that are commonly used in preclinical studies.

Many hepatotoxicants exert their toxicity through CRMs, which activate cellular stress responses, making stress response markers attractive as early indicators of CRM-mediated DILI in preclinical studies (Monroe et al. 2020; Guo and van den Beucken 2024). However, the translatability of transcriptional biomarker panels across preclinical models and species remains unclear. Morgenstern et al. (2024) identified 6 NRF2 activity markers conserved in humans, mice, and rats—*NQO1*, *TXNRD1*, *SRXN1*, *HMOX1*, *GCLC*, and *GCLM*. Our findings further support the robustness of 3 of these genes (*NQO1*, *TXNRD1*, and *SRXN1*) as translatable NRF2 activation biomarkers across a wider range of preclinical species. In contrast, no similar attempts have been made to establish a translatable panel of UPR activity biomarkers. The use of a panel of UPR-related genes as prognostic biomarkers in acute myeloid leukemia was recently reported (Fei et al. 2025). However, aberrant activation of the UPR in leukemia may render this panel inappropriate for the prediction of DILI potential in normal cells, whilst the wider species relevance of the panel is unclear. To address this gap, we leveraged RNA-seq data to identify 4 genes (*EIF2AK2*, *CHAC1*, *SLC40A1*, and *ASAP3*) commonly changed across humans and all preclinical species, representing a candidate panel of translatable UPR markers. Although further validation is required to assess the suitability of these biomarkers in relevant preclinical models, such panels may prove valuable in early hepatocyte-based *in vitro* screens for CRM liability in new drug candidates.

Although the models employed in this study—cultured primary hepatocytes and *ex vivo* liver tissue from all species of interest—were selected to best approximate *in vivo* hepatic physiology, several limitations should be considered. Human liver samples were obtained from clinically acceptable margins surrounding cancerous tissue (see Table S2 for donor details). As liver samples from healthy, cancer-free donors were unavailable, tissue distal to tumors was considered a surrogate for noncancerous tissue. Although this is consistent with established practice in studies utilizing *ex vivo* human liver tissue, the presence of preneoplastic cells or associated mutations, and any effects on stress response pathway components, in these samples cannot be fully excluded. On the other hand, most donors from whom cryopreserved hepatocytes were obtained (see Table S3 for donor details) underwent liver resections due to nonprimary liver cancers, lessening the likelihood of preneoplastic hepatocellular mutations in these samples. Cryopreserved hepatocytes were sourced from multiple commercial suppliers, each of whom use proprietary protocols for handling, isolation, and cryopreservation of hepatocytes, whereas isolation methods may also differ between species (i.e. *in situ* perfusion of rodent liver vs. *ex vivo* perfusion of nonrodent liver specimens). Neither aspect can be fully controlled by the end user, representing a potential source of variability between species. Moreover, there is potential for notable inter-donor variability, particularly for human hepatocytes, associated with the genetic and lifestyle characteristics of the donors and the quality of the liver tissue from which the cells are isolated (Table S3). Another limitation of this study is the use of thapsigargin at a concentration that displayed species-specific cytotoxicity against beagle hepatocytes, which may confound interpretation of their comparatively limited transcriptional response to ER stress. Finally, although it has been observed that female rodents typically display greater basal and adaptive antioxidant capacities than male animals (Russomanno et al. 2026), our study does not fully capture the contribution of sex to the observed inter-species differences in stress response pathway capacities due to the use of rodent hepatocytes from exclusively male donors. Given the increasing regulatory emphasis on the inclusion of both sexes in preclinical studies to maximally capture biological variation (Miller et al. 2017), future work should ensure both sexes are represented equally.

Overall, this study highlights notable intrinsic species-specific differences in a factor known to influence the progression of DILI, which should be accounted for during species selection to maximize the translatability of preclinical toxicology studies. Consideration of the enhanced hepatic capacity of rodents to mitigate cellular stress and chemical insult, which may underlie a relative resistance of these species to CRM-mediated DILI (Xu et al. 2021; Russomanno et al. 2023), could contribute to improving the predictive capabilities of preclinical *in vivo* toxicology studies. However, as a complete move away from poorly predictive rodents to more predictive nonrodent species is infeasible due to ethical and practical considerations that must be made during species selection (Son et al. 2020), our findings also reinforce the need to reduce reliance on animal models—which will always possess the potential to display species-specific differences to humans—in drug safety evaluation. This transition is encouraged by amendments to legislation and regulatory guidance, such as the US Food and Drug Administration (FDA) Modernization Act 2.0, which eliminated the legal requirement for animal toxicology studies to support new drug applications submitted to the FDA. The aspiration is that, as animal-free NAMs are increasingly validated and integrated into regulatory frameworks, they will ultimately replace traditional *in vivo* animal toxicology studies, providing more human-relevant, predictive approaches for evaluating drug safety during preclinical development.

## Supplementary Material

kfag061_Supplementary_Data
