# Supplementary material for: Hepatic cellular stress response pathways exhibit species differences in basal and inducible activity
Source: Toxicol Sci. 2026 May 28;209(6):kfag061. doi: 10.1093/toxsci/kfag061 (PMC13387358; doi:10.1093/toxsci/kfag061)
Supplement: kfag061_Supplementary_Data [file kfag061_supplementary_data.docx]

Supplementary Table 1. Liver tissue sample information.
*See Supplementary Table 2 for additional details of human liver tissue donors.

| **Species** | **Treatment** | **Sex & number of samples** | **Age at collection** | **Source of tissue** |
| --- | --- | --- | --- | --- |
| **Human*** | Planned partial hepatectomy, no neoadjuvant chemotherapy | Male (n=6)  Female (n=5) | 31-82 years | Aintree University Hospital |
| **CD1 mouse** | Untreated | Male (n=4) | 6-8 weeks | University of Liverpool |
| **C57BL/6 mouse** | Untreated | Male (n=4) | 9-10 weeks | University of Liverpool |
| **Sprague-Dawley rat** | Untreated | Male (n=4) | 9-10 weeks | University of Liverpool |
| **Wistar rat** | Untreated | Male (n=4) | 7-10 weeks | University of Liverpool |
| **Cynomolgus macaque** | 0.5% methylcellulose (4000 cP) in purified water, daily oral gavage, 28 days | Male (n=2)  Female (n=2) | 28-37 months | Charles River Laboratories |
| **Beagle dog** | Deionised purified water, pH adjusted to 3.5-4 with 1 M HCl, daily oral gavage, 28 days | Male (n=2)  Female (n=2) | 10-11 months | Charles River Laboratories |

Supplementary Table 2. Characteristics of human liver tissue donors.

| **Donor** | **Sex** | **Age** | **Diagnosis** |
| --- | --- | --- | --- |
| 1 | Male | 82 | Hepatocellular carcinoma |
| 2 | Female | 79 | *Unknown* |
| 8 | Male | 56 | Hepatocellular carcinoma |
| 12 | Female | 30 | Adenocarcinoma |
| 14 | Female | 76 | Hepatocellular carcinoma |
| 21 | Female | 73 | Hepatocellular carcinoma |
| 28 | Female | 47 | Hepatocellular carcinoma |
| 42 | Male | 31 | Hepatocellular carcinoma |
| 52 | Male | 68 | Hepatocellular carcinoma |
| 58 | Male | 65 | *Unknown* |
| 71 | Male | 63 | *Unknown* |

Supplementary Table 3. Commercial cryopreserved human hepatocyte information.

| **Sex** | **Age** | **Diagnosis** | **Supplier post-thaw viability** | **Observed post-thaw viability** |
| --- | --- | --- | --- | --- |
| Male | 64 | Colorectal liver metastases | 83% | >75% |
| Male | 74 | Colorectal liver metastases | 83% | >78% |
| Male | 51 | Hepatocellular carcinoma | 90% | >82% |

Supplementary Table 4. Western blot antibody information.
HRP, horseradish peroxidase.

| **Protein** | **Antibody supplier & code** | **Molecular weight (kDa)** | **Dilution** | **Host species** |
| --- | --- | --- | --- | --- |
| **β-Actin** | Abcam (ab6276) | 42 | 1:10,000 | Mouse |
| **TXNRD1** | Abcam (ab124954) | 55 | 1:5000 | Rabbit |
| **NQO1** | Abcam (ab2346) | 30 | 1:2000 | Goat |
| **GCLC** | Abcam (ab41463) | 73 | 1:5000 | Rabbit |
| **GCLM** | Abcam (ab126704) | 31 | 1:5000 | Rabbit |
| **BiP** | Proteintech (11587-1-AP) | 78 | 1:5000 | Rabbit |
| **sXBP1** | Proteintech (24868-1-AP) | 70 | 1:2000 | Rabbit |
| **SQSTM1** | Sigma (P0067) | 62 | 1:2500 | Rabbit |
| **LC3B** | Cell Signaling Technology (2775) | 14 & 16 | 1:750 | Rabbit |
| **HRP-conjugated anti-mouse IgG** | Sigma-Aldrich (A9044) | NA | 1:5,000 | NA |
| **HRP-conjugated anti-rabbit IgG** | Sigma-Aldrich (A9169) | NA | 1:5,000 | NA |
| **HRP-conjugated anti-goat IgG** | Dako (P0449) | NA | 1:5,000 | NA |

Supplementary Table 5. Primary antibody immunogen homology across species of interest.
aa, amino acid; H, human; M, mouse; R, rat; Mo, monkey (any species); D, dog; NA, not applicable.

|  | | | **% Immunogen homology (UniProt ID / NCBI Reference Sequence)** | | | | |
| --- | --- | --- | --- | --- | --- | --- | --- |
| **Protein** | **Supplier tested reactivity** | **Immunogen** | **Human** | **Mouse** | **Rat** | **Cynomolgus macaque** | **Dog** |
| **β-Actin** | H, M, R, Mo, D | Synthetic peptide corresponding to N-terminal of β-Actin: DDDIAALVIDNGSGK | 92.9 (Q9UMN3) | 92.9 (P60710) | 92.9 (P60711) | 92.9 (Q4R561) | 92.9 (O18840) |
| **TXNRD1** | H, M, R | Recombinant peptide corresponding to aa 1-613 of mouse TXNRD1 (UniProt Q9JMH6): MPVDDCWLYFPASRGRTFVQTVWVAPTCPNCCWFPGFLPPVPRPPHVPRVLLRGPRGAVLPASRPSKTLPSSSQTPCPTDPCICPPPSTPDSRQEKNTQSELPNKKGQLQKLPTMNGSKDPPGSYDFDLIIIGGGSGGLAAAKEAAKFDKKVLVLDFVTPTPLGTRWGLGGTCVNVGCIPKKLMHQAALLGQALKDSRNYGWKVEDTVKHDWEKMTESVQSHIGSLNWGYRVALREKKVVYENAYGRFIGPHRIVATNNKGKEKIYSAERFLIATGERPRYLGIPGDKEYCISSDDLFSLPYCPGKTLVVGASYVALECAGFLAGIGLDVTVMVRSILLRGFDQDMANKIGEHMEEHGIKFIRQFVPTKIEQIEAGTPGRLRVTAQSTNSEETIEGEFNTVLLAVGRDSCTRTIGLETVGVKINEKTGKIPVTDEEQTNVPYIYAIGDILEGKLELTPVAIQAGRLLAQRLYGGSNVKCDYDNVPTTVFTPLEYGCCGLSEEKAVEKFGEENIEVYHSFFWPLEWTVPSRDNNKCYAKIICNLKDDERVVGFHVLGPNAGEVTQGFAAALKCGLTKQQLDSTIGIHPVCAEIFTTLSVTKRSGGDILQSGCUG | 90.8 (Q16881) | 100 (Q9JMH6) | 96.1 (O89049) | 90.9 (A0A2K5VHI7) | 89.4 (A0A8P0T4C1) |
| **NQO1** | H, R | Synthetic peptide corresponding to aa 267-274 (C-terminal) of human NQO1 (UniProt P15559): SIPTDNQIKARK | 100 (P15559) | 91.7 (Q64669) | 91.7 (P05982) | 100 (A0A2K5TZM3) | 100 (A0A8I3NAN7) |
| **GCLC** | H | Synthetic peptide corresponding to aa 50-100 of human GCLC (UniProt P48506). Inferred immunogen: EVEYMLVSFDHENKKVRLVLSGEKVLETLQEKGERTNPNHPTLWRPEYGSYMIEGTPGQPYGGTMSEFNTVEANMRKRRKEATSILEENQALCTITSFPRL | 100 (P48506) | 91.1 (P97494) | 90.1 (P19468) | 99.0 (Q2PFQ9) | 96.0 (A0A8I3NES9) |
| **GCLM** | H | Synthetic peptide corresponding to aa 50-100 of human GCLM (UniProt P48507). Inferred immunogen: LNEWSSQINPDLVREFPDVLECTVSHAVEKINPDEREEMKVSAKLFIVESNSSSSTRSAVDMACSVLGVAQLDSVIIASPPIEDGVNLSLEHLQPYWEEL | 100 (P48507) | 96.0 (O09172) | 95.0 (P48508) | 99.0 (G7NTP5) | 97.0 (A0A8I3P689) |
| **BiP** | H, M, R | Purified peptide corresponding to aa 351-654 of human BiP (UniProt P11021): LKKSDIDEIVLVGGSTRIPKIQQLVKEFFNGKEPSRGINPDEAVAYGAAVQAGVLSGDQDTGDLVLLDVCPLTLGIETVGGVMTKLIPRNTVVPTKKSQIFSTASDNQPTVTIKVYEGERPLTKDNHLLGTFDLTGIPPAPRGVPQIEVTFEIDVNGILRVTAEDKGTGNKNKITITNDQNRLTPEEIERMVNDAEKFAEEDKKLKERIDTRNELESYAYSLKNQIGDKEKLGGKLSSEDKETMEKAVEEKIEWLESHQDADIEDFKAKKKELEEIVQPIISKLYGSAGPPPTGEEDTAEKDEL | 100 (P01130) | 99.2 (P20029) | 98.8 (P06761) | 100 (A0A2K5WXY8) | 98.4 (A0A8C0MIT4) |
| **sXBP1** | H, M, R | Purified peptide corresponding to aa 167-376 of human XBP1 (UniProt P17861): GAGPVVTPPEHLPMDSGGIDSSDSESDILLGILDNLDPVMFFKCPSPEPASLEELPEVYPEGPSSLPASLSLSVGTSSAKLEAINELIRFDHIYTKPLVLEIPSETESQANVVVKIEEAPLSPSENDHPEFIVSVKEEPVEDDLVPELGISNLLSSSHCPKPSSCLLDAYSDCGYGGSLSPFSDMSSLLGVNHSWEDTFANELFPQLISV | 100 (P17861) | 88.4 (O35426) | 87.4 (Q9R1S4) | 98.6 (XP_015312823.1) | 88.1 (A0A8I3QXH5) |
| **SQSTM1** | H, M, R | Synthetic peptide corresponding to aa 56-269 of human SQSTM1 (UniProt Q13501). Inferred immunogen: IDVEHGGKRSRLT | 100 (Q13501) | 100 (Q64337) | 100 (O08623) | 100 (A0A7N9CMX6) | 100 (A0A8C0QJ48) |
| **LC3B** | H, M, R | Synthetic peptide corresponding to aa 1-125 of human LC3B (UniProt Q9GZQ8). Inferred immunogen: MPSEKTFKQRRTFEQRVEDVRLIREQHPTKIPVIIERYKGEKQLPVLDKTKFLVPDHVNMSELIKIIRRRLQLNANQAFFLLVNGHSMVSVSTPISEVYESEKDEDGFLYMVYASQETFGMKLSV | 100 (Q9GZQ8) | 95.2 (Q9CQV6) | 96.0 (Q62625) | 100 (XP_045239064.1) | 96.6 (XP_038394024.1) |


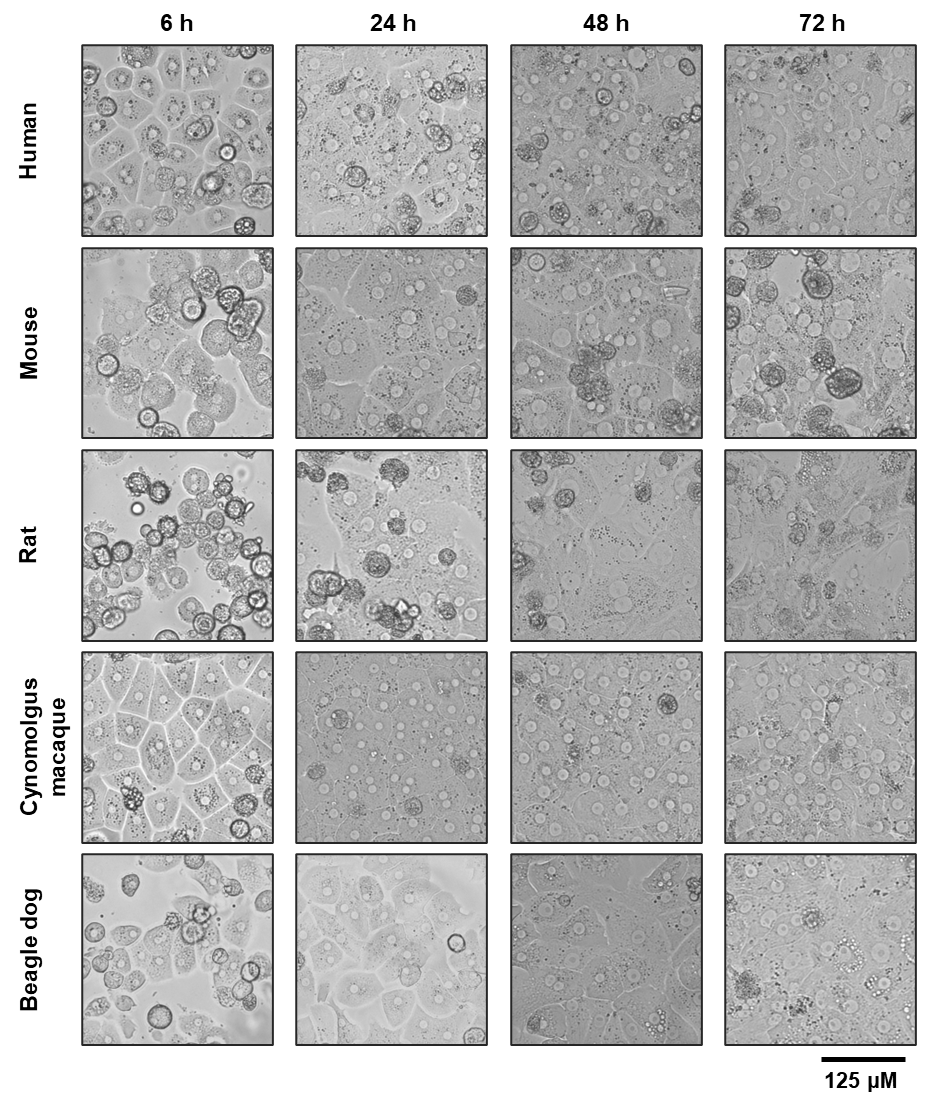


Supplementary Figure 1. Primary hepatocytes cultured in serum-supplemented medium retain their morphology for at least 48 h in vitro.
Morphology of human, CD1 mouse, Sprague-Dawley rat, cynomolgus macaque and beagle dog primary hepatocytes cultured in serum-supplemented hepatocyte culture medium 6, 24, 48 and 72 h post-plating in 2D monolayer culture on a collagen I-coated plated.


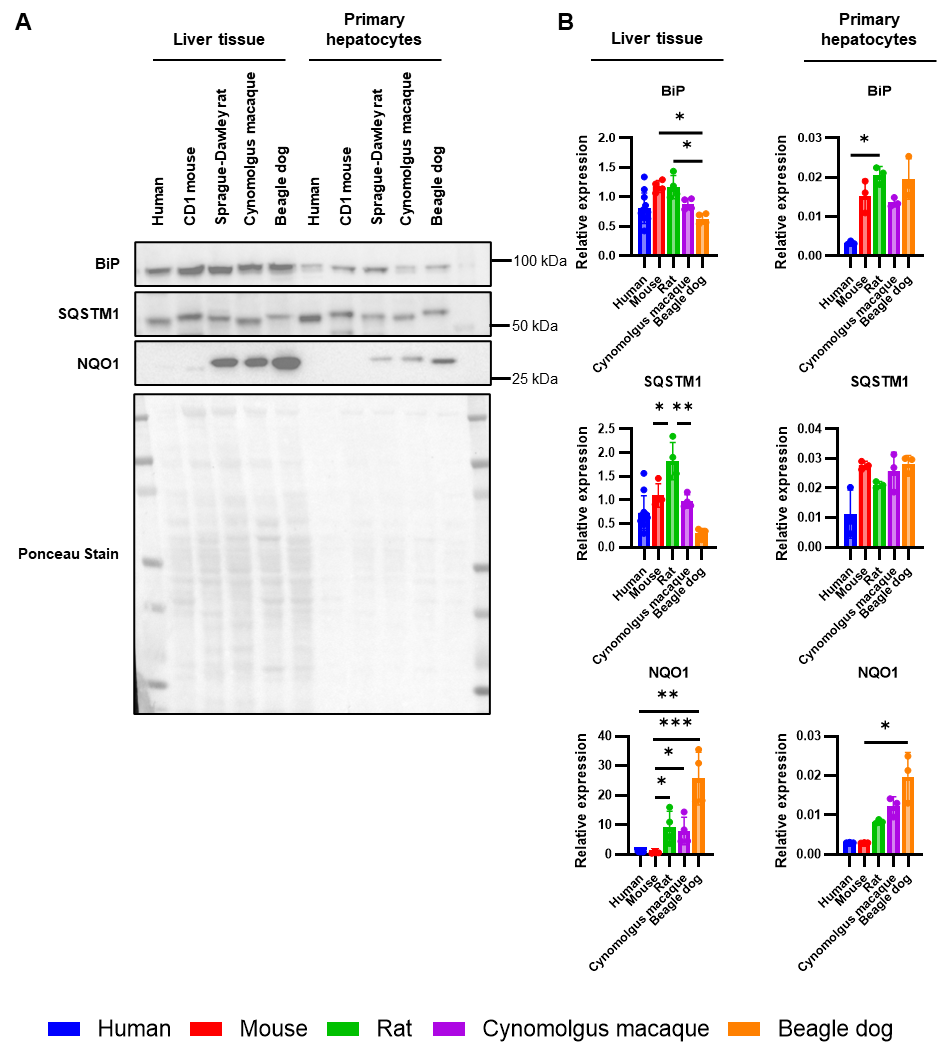


Supplementary Figure 2. Verification of species differences across *ex vivo* and *in vitro* liver models.
(A) Western blot visualization and (B) densitometric quantification of the basal expression of the stress response-associated proteins BiP, SQSTM1 and NQO1 in snap-frozen *ex vivo* liver tissue (n=11 human, n=4 all other species) and primary hepatocytes cultured for 24 h (n=3/species). Target protein expression was normalized to total protein expression. Data represent mean ± standard deviation. Inter-species comparisons performed using a Kruskal-Wallis test followed by Dunn’s post-hoc test. Significant inter-species comparisons indicated on the graph: * *p*_adj_ ≤ 0.05, ** *p*_adj_ ≤ 0.01, *** *p*_adj_ ≤ 0.001.


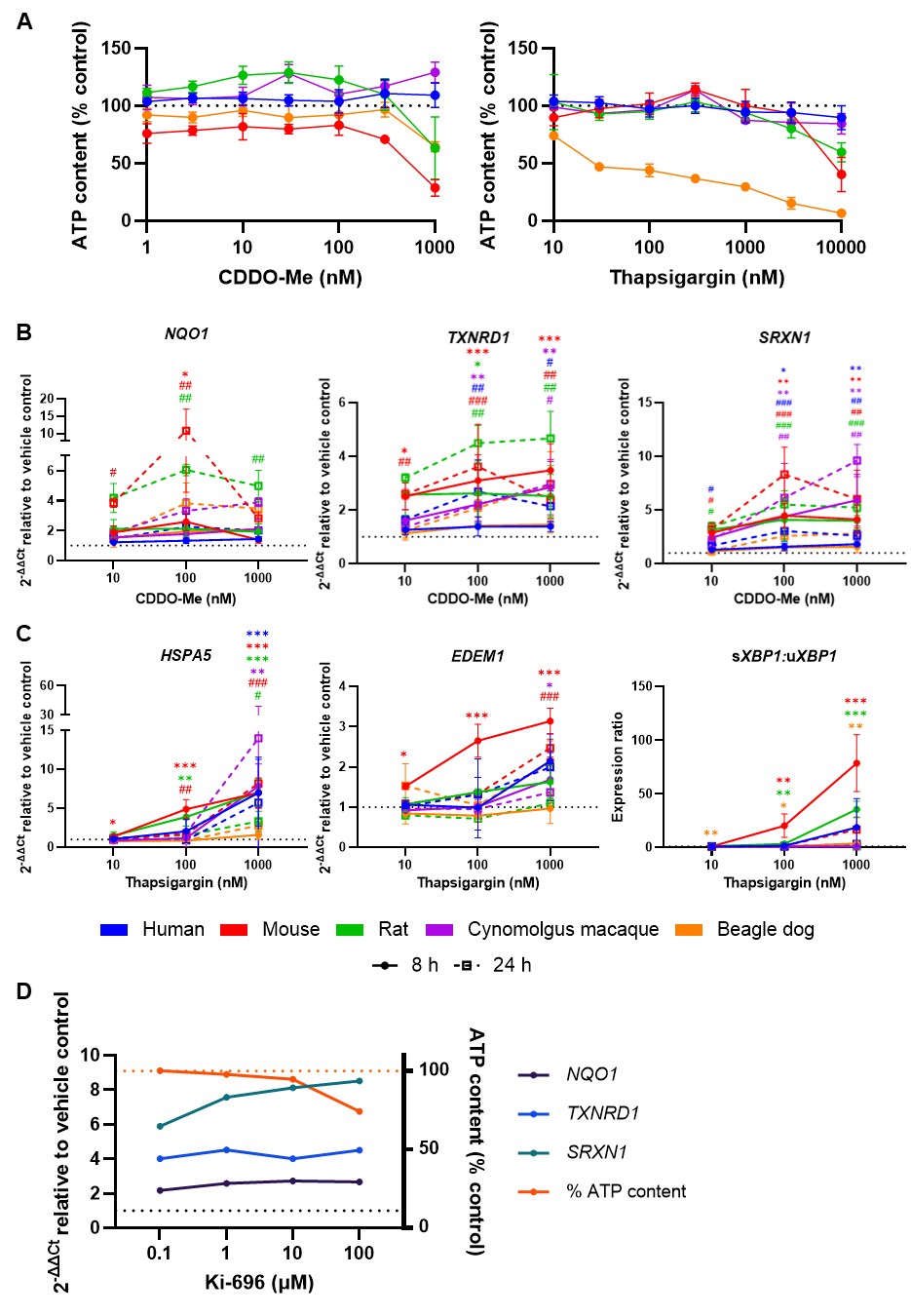


Supplementary Figure 3. Selection of concentrations for NRF2 pathway and UPR modulators.
(A) Measurement of the effect of 24 h exposure to CDDO-Me or thapsigargin, relative to 0.5 % (v/v) DMSO vehicle control, on ATP content of primary hepatocytes. Data represent mean ± standard deviation of n=2 biological replicates. RT-qPCR analysis of the expression of (B) NRF2 target genes or (B) UPR target genes following 8 h or 24 h exposure to varying concentrations of a pathway modulator. Data represent mean ± standard deviation of three biological replicates (n=3) of 2^-ΔΔCt^ of target gene relative to 0.5 % (v/v) DMSO vehicle control and normalized against expression of the housekeeping genes GAPDH and ACTB. Significant comparisons to vehicle control, calculated by one-way ANOVA and Tukey’s post-hoc test or Kruskal-Wallis test and Conover-Iman post-hoc test, as appropriate, are indicated on the graph. Asterisks denote significant comparisons at 8 h treatment, hashes denote significant comparisons at 24 h treatment; */# p_adj_ ≤ 0.05, **/## p_adj_ ≤ 0.01, ***/### p_adj_ ≤ 0.001. (D) Assessment of cellular ATP content or NRF2-associated gene expression in HepG2 cells following exposure to varying concentrations of Ki696 for 24 h. Data represent one biological replicate (n=1) of (left y-axis) 2^-ΔΔCt^ of target gene relative to 0.5% (v/v) DMSO vehicle control and normalized against expression of the housekeeping genes GAPDH and ACTB or (right y-axis) cellular ATP content relative to 0.5 % (v/v) DMSO vehicle control. Black dotted line indicates mRNA expression in vehicle control-treated cells. Orange dotted line represents ATP content of vehicle control-treated cells.


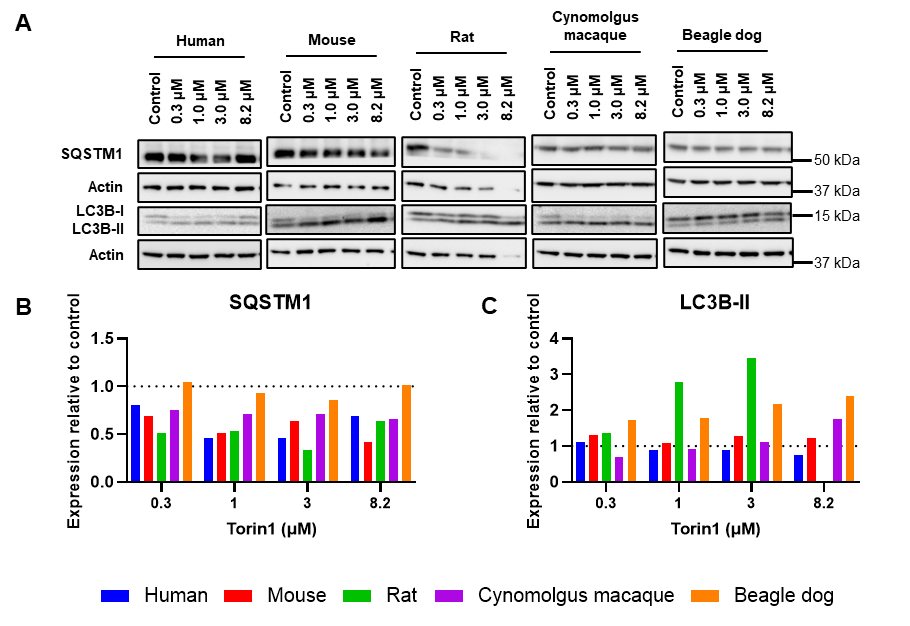


Supplementary Figure 4. Autophagy activator screen.
(A) Western blot visualization and densitometric quantification of the expression of autophagy-associated proteins (B) SQSTM1 and (C) LC3B-II in primary hepatocytes following 4 h exposure to various concentrations of the autophagy activator Torin1 (0.3 – 8.2 µM). All data represent n=1 of target protein expression normalized against expression of the housekeeping protein β-actin and relative to 0.5 % (v/v) vehicle control.


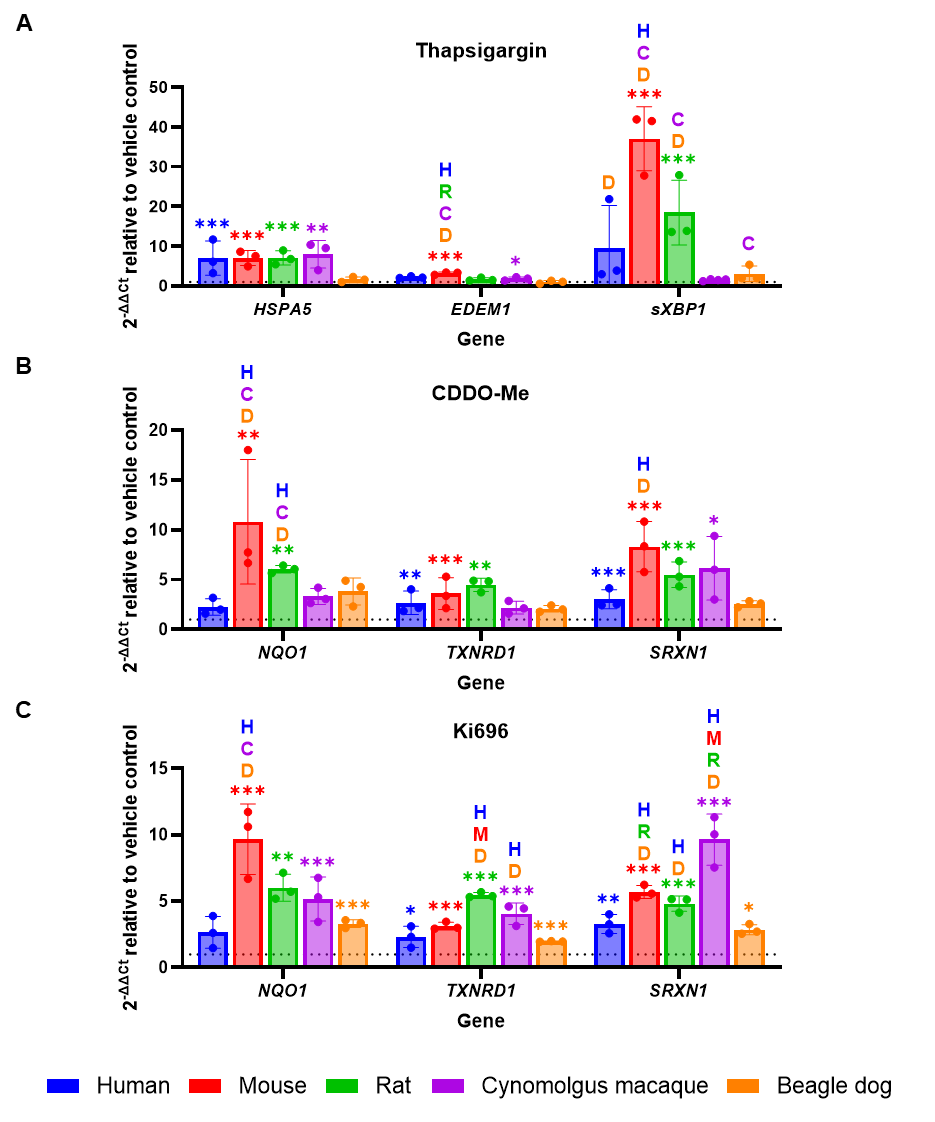


Supplementary Figure 5. Targeted assessment of stress response induction.
Induction of mRNA targets of (A) the UPR or (B & C) the NRF2 pathway in primary hepatocytes by (A) thapsigargin (1 μM, 8 h), (B) CDDO-Me (100 nM, 24 h), or (C) Ki696 (10 μM, 24 h), measured by RT-qPCR. All data represent mean ± standard deviation of three biological replicates (n=3) of 2^-ΔΔCt^ of target gene relative to vehicle control and normalized against expression of the housekeeping genes GAPDH and ACTB. Significant differences relative to vehicle control and between species assessed using a One-Way ANOVA and Tukey’s post-hoc test or Kruskal-Wallis and Dunn’s post-hoc test, as appropriate. Asterisks denote significant changes relative to vehicle control; * p_adj_ ≤ 0.05, ** p_adj_ ≤ 0.01; *** p_adj_ ≤ 0.001. Letters denote significantly greater induction than the indicated species (p_adj_ ≤ 0.05): H, human; M, mouse; R, rat; C, cynomolgus macaque; D, beagle dog.


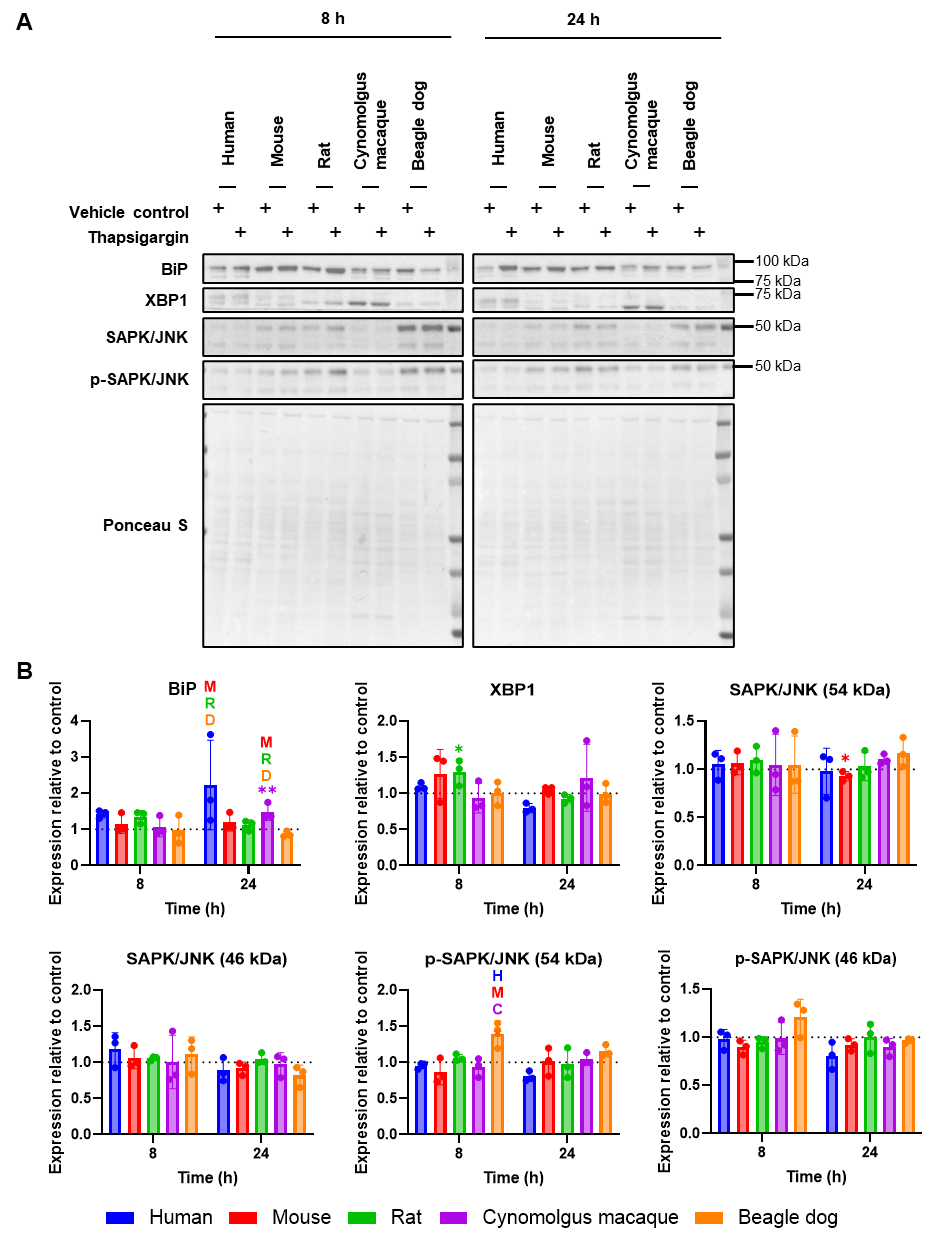


Supplementary Figure 6. Effects of thapsigargin on UPR protein expression in primary hepatocytes.
(A) Western blot visualization and (B) densitometric quantification of UPR-associated protein expression in primary hepatocytes following exposure to 1 μM thapsigargin, or 0.5 % (v/v) DMSO vehicle control, for 8 or 24 h. Ponceau S stain loading control shown. Samples representative of each group were generated by pooling equal amounts of protein from each biological replicate sample (n=3). All data represent mean ± standard deviation of three biological replicates (n=3) of target protein expression relative to total protein expression (Ponceau S signal) and normalized to vehicle control. Differences in protein level relative to vehicle control were assessed using an unpaired Student’s t-test or Mann-Whitney U test, while inter-species differences were assessed using a one-way ANOVA and Tukey’s post-hoc test or Kruskal-Wallis test and Conover-Iman post-hoc test. Asterisks denote significant changes relative to vehicle control; * p_adj_ ≤ 0.05, ** p_adj_ ≤ 0.01. Letters denote significantly greater induction than the indicated species (p_adj_ ≤ 0.05): H, human; M, mouse; R, rat; C, cynomolgus macaque; D, beagle dog.


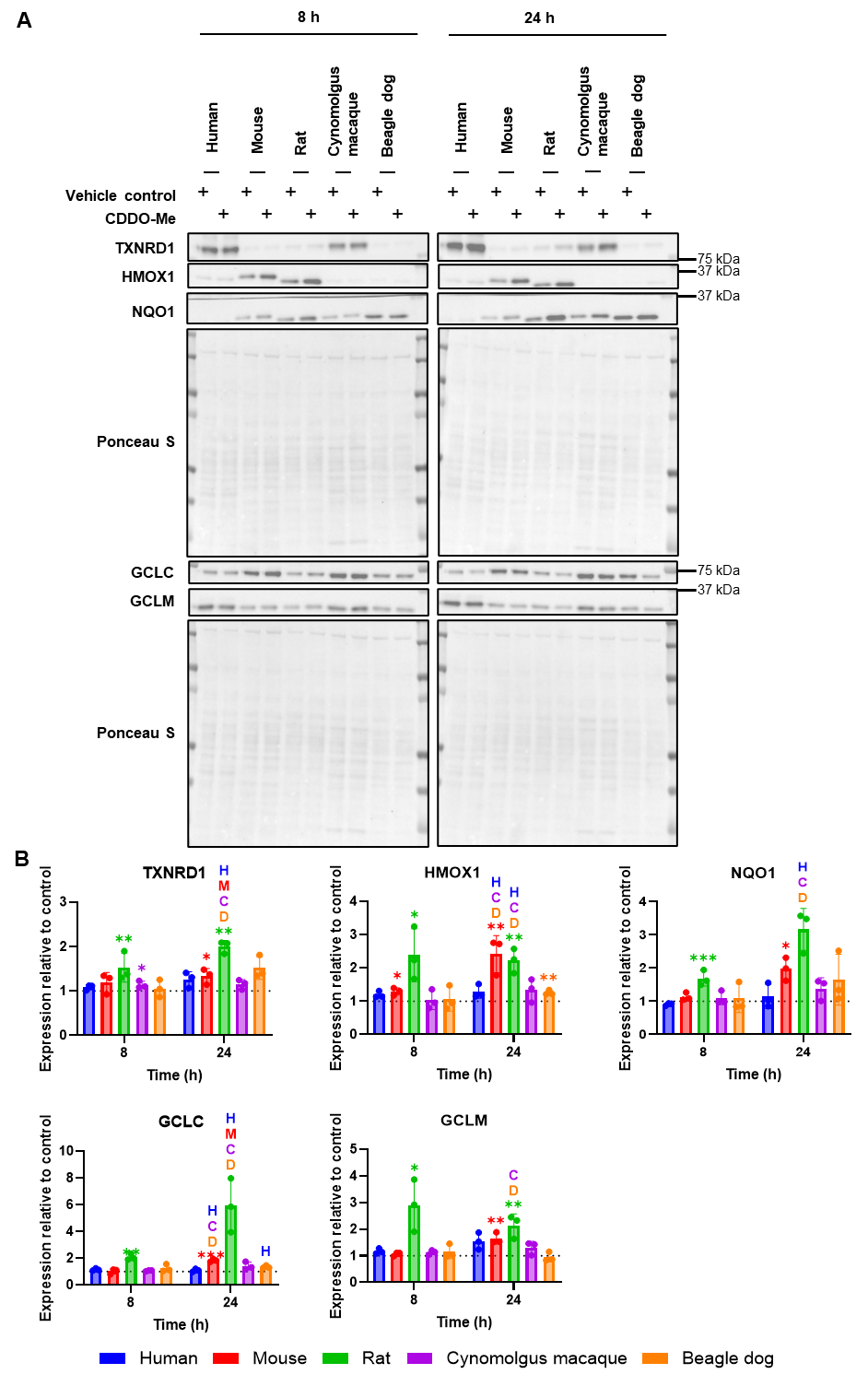


Supplementary Figure 7. The effects of CDDO-Me on NRF2 pathway proteins in primary hepatocytes.
(A) Western blot visualization and (B) densitometric quantification of NRF2 pathway target proteins in primary hepatocytes following exposure to 100 nM CDDO-Me, or 0.5 % (v/v) DMSO vehicle control, for 8 or 24 h. Ponceau S stain loading control shown. Samples representative of each group were generated by pooling equal amounts of protein from each biological replicate sample (n=3). All data represent mean ± standard deviation of three biological replicates (n=3) of target protein expression relative to total protein expression (Ponceau S signal) and normalized to vehicle control. Differences in protein induction relative to vehicle control were assessed using an unpaired Student’s t-test or Mann-Whitney U test, while inter-species differences were assessed using a one-way ANOVA and Tukey’s post-hoc test or Kruskal-Wallis test and Conover-Iman post-hoc test. Asterisks denote significant changes relative to vehicle control; * p_adj_ ≤ 0.05, ** p_adj_ ≤ 0.01, *** p_adj_ ≤ 0.001. Letters denote significantly greater induction than the indicated species (p_adj_ ≤ 0.05): H, human; M, mouse; C, cynomolgus macaque; D, beagle dog.

Supplementary Files 1-6 can be accessed at <https://doi.org/10.5281/zenodo.18335533>.
